# A conceptual model for dissolved P mobilization from legacy sources

**DOI:** 10.1002/jeq2.70003

**Published:** 2025-02-20

**Authors:** D. M. Nash, R. W. Mc Dowell, P. J. A. Kleinman, P. A. Moore, J. M. Duncan, P. M. Haygarth, D. R. Smith, A. Iho

**Affiliations:** ^1^ School of Agriculture and Food, Faculty of Veterinary and Agricultural Sciences The University of Melbourne Parkville Victoria Australia; ^2^ Soil and Allied Services Pty. Ltd. Port Welshpool Victoria Australia; ^3^ AgResearch, Lincoln Science Centre Christchurch New Zealand; ^4^ Faculty of Agriculture and Life Sciences Lincoln University Christchurch New Zealand; ^5^ USDA‐ARS Soil Management and Sugarbeet Research Laboratory Fort Collins Colorado USA; ^6^ USDA‐ARS Poultry Production and Product Safety Research Laboratory Fayetteville Arkansas USA; ^7^ Department of Ecosystem Science & Management The Pennsylvania State University University Park Pennsylvania USA; ^8^ Lancaster Environment Centre Lancaster University Bailrigg UK; ^9^ USDA‐ARS Grassland Soil and Water Research Laboratory Temple Texas USA; ^10^ Faculty of Social Science & Business Studies University of Eastern Finland Joensuu Finland

## Abstract

Excessive phosphorus (P) concentrations can lead to conditions that limit the amenity of freshwater resources. This problem is particularly acute in agricultural catchments, where P fertilizer and manure amendments have been used to increase soil fertility and productivity. In these catchments, P indices are often used to help target critical source areas in order to reduce P exports. However, the overall impact of agricultural mitigation efforts on receiving waters has not always been consistent with declines in total P exports from catchments. In this paper we propose a model of dissolved P mobilization (i.e., entrainment) in surface runoff that accounts for this outcome and examine modifications to P indices that better accommodate dissolved P mobilization. We suggest that dissolved P mobilization commences near the soil surface and has two phases. When water is first applied, labile P is mostly mobilized by dissolution and advection. Subsequently, as the supply of readily accessible P is exhausted, diffusion and hydrodynamic dispersion mobilize P from other sources at a near constant rate for the remainder of the event. As most P exports occur in larger (i.e., longer) events, the second phase appears responsible for most dissolved P exports. Such a model of dissolved P mobilization is consistent with runoff monitoring data under natural and simulated rainfall, suggesting that on low (shallow) slopes where the interaction between surface soil and water may be prolonged, dissolved P concentrations are likely to be higher. Dissolved P mobilization from low‐slope areas is not well represented in P indices at present. We suggest that there needs to be a more complex, mechanistic structure to P indices that involves additional compartmentalization. Further, we suggest that this can be achieved without losing the simplicity of P indices or flexibility to integrate research data and experiential knowledge into tools that are relevant to specific regions.

## Core Ideas

- Dissolved P exports from agriculture are mobilized at the soil surface.
- We propose a conceptual model of dissolved P mobilization from legacy sources.
- Using that model, we review P indices and their applicability to dissolved P exports from legacy P.
- Modifications to P indices are suggested.

**Abbreviations**
CSA: critical source area
DP: dissolved phosphorus
DRP: dissolved reactive phosphorus
STP: soil test phosphorus
TP: total phosphorus

## 1 INTRODUCTION

Exports of (P) from land to water in drainage are a natural consequence of any terrestrial plant production system. In many countries those exports lead to conditions that limit the amenity of freshwater resources. Exports of P from land to water are a particular problem in agricultural catchments, where P amendments have been used to stimulate agricultural production (Grayson et al., 2001; McDowell et al., 2019; Pierson et al., 2001; Sharpley et al., 2007; Sharpley, Richards, et al., 2012).

The challenge in reducing P exported from agricultural catchments is evident from water quality targets. The total phosphorus (TP) concentrations designed to minimize adverse algal (viz. periphyton) growth are commonly <0.009–0.07 mg/L, varying by region or jurisdiction (ANZECC & ARMCANZ, 2018; CCME, 2004; EPA, 2003; Miltner, 2010; Smith & Tran, 2010; Stevenson et al., 2008). In comparison, the TP concentration in soil water for optimum crop growth is often cited as >0.2 mg/L (Pierzynski et al., 2005). Moreover, a soil water P concentration of 0.9 mg/L was required for maximum pasture productivity in a New Zealand study (Wheeler & Edmeades, 1995), and concentrations >1 mg/L have been noted in Australia (Nash et al., 2007; Toifl et al., 2000; Watkins et al., 2012).

Phosphorus is exported from land to water as part of a solid (e.g., mineral P compounds or soil aggregates), attached to a solid (e.g., adsorbed P), or as a solute (i.e., dissolved in water). The terms “dissolved” and “particulate” are commonly used to define P materials that pass through or are retained by a 0.45 µm filter. Particulate P (PP; >0.45 µm) comprises crystalline P, adsorbed P, and P in organic matter. Dissolved phosphorus (DP; <0.45 µm) on the other hand is often dominated by inorganic orthophosphate (H_2_PO_4_^−^/HPO_4_^2−^), the form of P utilized by both terrestrial and aquatic organisms (Robinson et al., 1994; Walton & Lee, 1972). Dissolved P may also comprise P attached to, or contained in, colloidal materials that are <0.45 µm (Beckett & Hart, 1993; Haygarth & Sharpley, 2000; Haygarth et al., 1997), and some organic P species, with mono‐ and diphosphate esters most common (Kolowith et al., 2001). Being more bioavailable, DP has a greater impact on receiving waters than PP.

Given the difficulties in addressing P exports, considerable effort has been expended to investigate the possible effects of climate change on TP exports from agricultural catchments. For example, a global climate model (Collins et al., 2001; Gordon et al., 2000; Pope et al., 2000) and the soil and water assessment tool (Gassman et al., 2007; Yuan & Koropeckyj‐Cox, 2022), a distributed hydrological model, have been used to simulate P export processes in Chinese agriculture (Zhang et al., 2020). That study suggests that an increase in runoff caused by climate change will be accompanied by an increase in the export of TP. Similar outcomes have been predicted when high‐resolution catchment data, climate projections, and two models of contrasting complexity (i.e., a process‐based model and a data‐based mechanistic model) were used to investigate future scenarios for three agricultural catchments in the UK (Ockenden et al., 2017). There may be an increasing need to mitigate P exports from agriculture in the future.

In agricultural catchments, P may be derived from point sources (e.g., discharges of treated sewage and industrial effluents, urban dwellings) or diffuse sources (e.g., broad‐scale agriculture). Within the diffuse source category, specific behaviors can dramatically increase short‐term P exports. For this reason, P exports are considered to have “systematic” (i.e., base or background) and “incidental” (i.e., management) components (Haygarth & Jarvis, 1999). However, specific activities (or incidents) may contribute to both systematic and incidental P exports. For example, the inappropriate use of P amendments (e.g., P applications in excess of requirements, inappropriate application methods) can account for a substantial portion (30%–80%) of total farm P exports in the year of application. When appropriately managed, that figure can be <10% (Nash & Hannah, 2011; Nash et al., 2019). Importantly, ongoing P surpluses would be expected to gradually increase systematic exports by increasing P concentrations in soil (Condron, 2004; Gourley et al., 2012; Neville et al., 2005; Stutter et al., 2015; Tian et al., 2017). This so‐called “legacy P” (Kleinman et al., 2011) can represent a recalcitrant baseline underpinning systematic exports (Haygarth et al., 2014; Jarvie et al., 2013; Sharpley et al., 2013).

At the watershed or catchment scale, hydrological models paired with an algorithm that estimates P export risk (Davies et al., 2006; Donigian et al., 1984; Novotny et al., 1978) have been useful in identifying areas where P export mitigation should be prioritized (Grayson, 2006). At the sub‐catchment and farm scales, process‐based models should be ideal for analyzing, mitigating, and regulating P exports, including those originating from legacy P (Borah & Bera, 2004; Cichota & Snow, 2009; Kumaran et al., 2011). Unfortunately, there are rarely sufficient data to either adequately parameterize complex process models or provide confidence in their predictions (Vaze et al., 2011). Phosphorus indices are simpler, semiquantitative tools used to assess the risk of P loss from fields to surface waters based on simple arithmetic computations of source (e.g., soil test phosphorus [STP], P applications in manure and fertilizer) and transport factors (e.g., erosion, surface runoff, subsurface drainage, and connectivity) (Buczko & Kuchenbuch, 2007; Lemunyon & Gilbert, 1993). However, in some jurisdictions, there is increasing evidence that efforts to mitigate TP exports from agriculture based on these tools have not achieved the desired outcomes due to enhanced DP exports (Jarvie et al., 2017; Singh et al., 2023; Smith et al., 2018).

In this paper we present a conceptual model of DP mobilization from agricultural systems. That model is then used to examine why mitigation activities based on current P indices may inadvertently increase DP as a proportion of TP exports, especially where enhanced soil fertility is a legacy of persistent P surpluses. We suggest some changes to P indices that will help them better represent DP export potential.

**Core Ideas**

- Dissolved P exports from agriculture are mobilized at the soil surface.
- We propose a conceptual model of dissolved P mobilization from legacy sources.
- Using that model, we review P indices and their applicability to dissolved P exports from legacy P.
- Modifications to P indices are suggested.

## 2 A CONCEPTUAL MODEL OF DP MOBILIZATION

### 2.1 Where DP exports start

**FIGURE 1:**
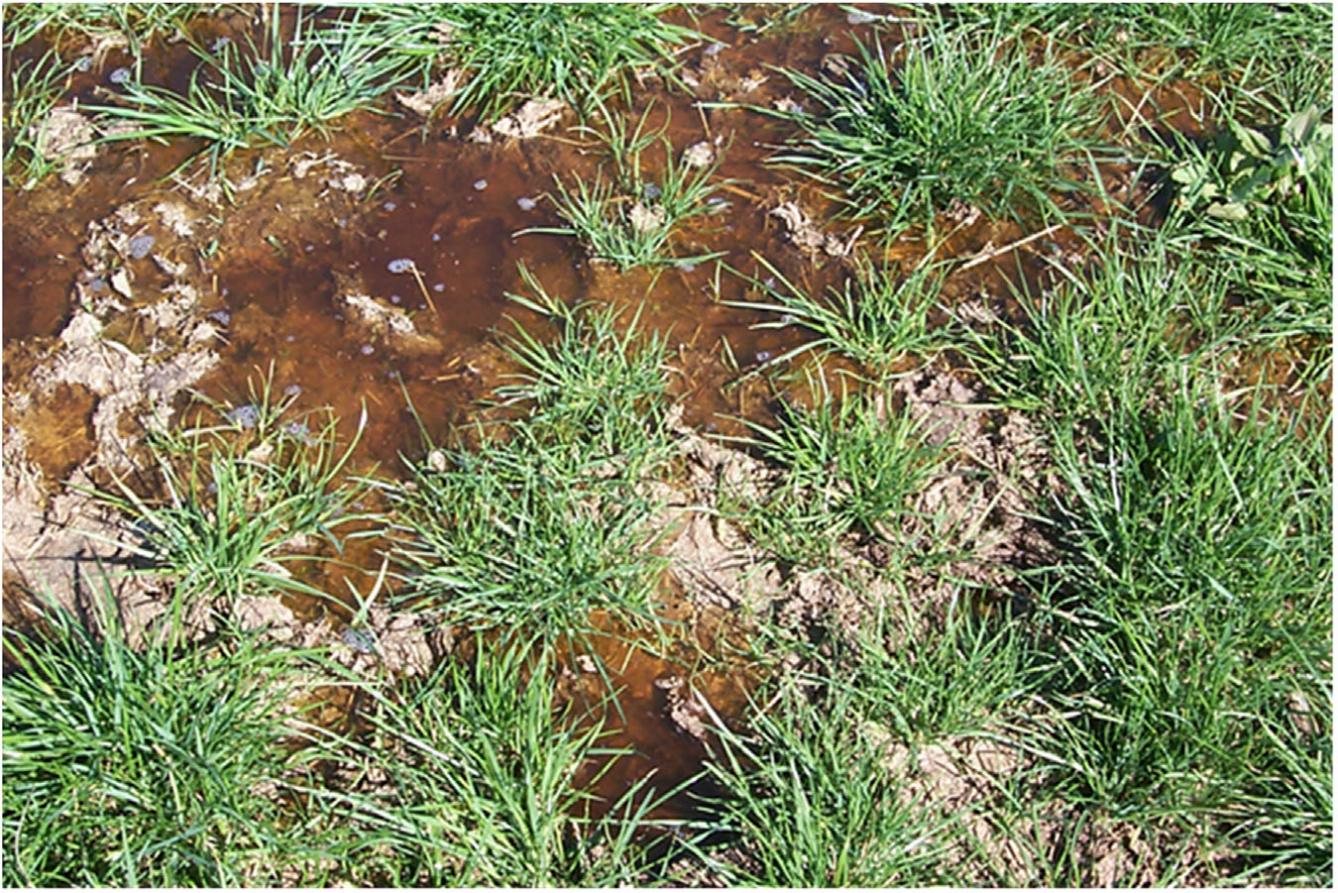
Overland flow on a dairy farm in south‐eastern Australia.

For P to be a water quality problem, there needs to be a source of P that is mobilized into water and transported to a location where its adverse effects are expressed (Haygarth et al., 2005). In the case of surface runoff, the problem starts when rain falls or irrigation water is applied to a field. It flows over and through vegetation and onto the soil surface. It is on the soil surface that animals defecate, plant detritus falls (see Figure 1), and P amendments are often applied. In both agricultural and natural systems, P concentrations are generally highest at the soil surface and decline with depth (i.e., P stratification occurs) (Baker et al., 2017; Cade‐Menun et al., 2015; Diaz‐Zorita & Grove, 2002; Dougherty et al., 2006; Osterholz et al., 2020; Robbins & Voss, 1991; Ryan et al., 2017; Saarela & Vuorinen, 2010; Weaver et al., 2023). It is in the soft (i.e., spongy) mixture of inorganic and organic materials at the soil surface that DP is first released from source materials and entrained into the water that will ultimately transport it offsite. While the processes responsible for the release of DP may vary (e.g., desorption, dissolution), it is the depth of the soil‐water interaction and quantity of P that is entrained that ultimately determine the quantity of DP available for export. In this paper the release and entrainment of DP will collectively be referred to as “DP mobilization.”

A study applying high‐intensity simulated rainfall (65 mm/h) on bare soil packed into boxes amended with ^32^P has demonstrated that the interaction between surface runoff and soil occurs within 3 mm of the surface (Ahuja et al., 1981). In many cases that is probably the maximum depth in which peak DP mobilization would be expected to occur. Natural rainfall is usually less intense than simulated rainfall, and, in agricultural soils, the plant canopy and surface detritus are likely to intercept raindrops. Both would be expected to lower the kinetic energy (i.e., impact energy) available for aggregate disruption and mixing (Sharpley, 1985), reducing the depth at which water and soil initially interact. For most practical purposes, it can be assumed that the layer of detritus (i.e., decomposing organic matter, including stover, thatch, fragments of dead organisms, and fecal material) and associated biological communities on or near the soil surface is probably a reasonable approximation for where most DP exports originate. That being said, in the field identifying where the transition from the detritus to the inorganic soil matrix occurs can be difficult, making numerical quantification of the pool of DP vulnerable to export prone to error.

The physical condition of surface soil can also influence DP mobilization. Animal traffic, machinery, and crop damage (e.g., dislodgement by wind) often increase the soil surface area and decrease soil infiltration rates (Drewry, 2006; Drewry et al., 2008; Greenwood & McKenzie, 2001). Such conditions facilitate water access to more surface soil and for longer periods (see Figure 1). It follows that the “effective” depth interaction (Ahuja et al., 1981) between water and soil is likely to vary both within and between production systems.

Live plants (McDowell et al., 2011; Sharpley, 1981; Timmons et al., 1970; Toor et al., 2003) and their residues (Noack et al., 2012) can be important sources of DP (Nash & Halliwell, 1999), especially after grazing (Mundy et al., 2003) or freeze‐thaw processes (Bechmann et al., 2005). The water‐soluble P and TP content of plants increase with soil fertility (Bromfield & Jones, 1972; Jones & Bromfield, 1969) and are therefore a legacy of previous P management. Plant exudates can contribute to a surface coating of dissolved reactive phosphorus (DRP) that can be removed by rainfall or irrigation water. However, P from inside both fallen and standing detritus and living organisms is more difficult to mobilize (Dougherty et al., 2004).

Inorganic minerals and adsorbed P are two other important sources of DP. In the absence of recently applied fertilizers or other P amendments (i.e., incidental effects), it would be expected that orthophosphate from these sources would be controlled by sorption/desorption and, to a lesser extent, dissolution processes (Barrow, 1983; Barrow, 1989, 2021; Holford, 1991; Norrish & Rosser, 1983; Penn & Camberato, 2019; Sample et al., 1980; Sanyal & DeDatta, 1991; Schulthess & Sparks, 1991). While chemical equilibria are unlikely to be consistent across a field (White & Beckett, 1964), the small variations in these processes across a year would not be expected to unduly affect DP mobilization. However, in the longer term, if P ultimately forms lower solubility precipitates, or through solid‐state diffusion penetrates the variable‐charge (e.g., changing with pH) surfaces of clay entities (Barrow, 1984), DP mobilization is likely to decline.

In many agricultural systems a significant proportion of soil P may be cycled through biological systems (McLaughlin et al., 1990; Nash et al., 2014; Perrott & Sarathchandra, 1989). The release of both inorganic and organic forms of DP from these systems would vary between seasons in response to substrate availability and biological activity (Bromfield & Jones, 1970; Harrison, 1987; Kaila, 1949; Magid & Nielson, 1992; Pote et al., 1999; Saunders & Metson, 1971; Shand et al., 1994). As a consequence, DP mobilization may be highest at times of the year when offsite drainage might be expected, for example, in early spring when temperatures rise or in summer when irrigation water is applied. Relationships between the tannin color, dissolved organic carbon, and DP in overland flow are consistent with the proposition (Halliwell et al., 2000; Nash et al., 2014) that organic materials make a substantial contribution to systematic (i.e., legacy) DP exports.

### 2.2 A model of DP mobilization

Historically, DP mobilization has been conceptualized as a “desorption” reaction that occurs in a finite volume of surface soil where mixing is facilitated by raindrop impact and flowing water (i.e., a mixing layer model) (Sharpley, 1985; Sharpley et al., 1981). It follows that DP concentrations could be estimated from the product of “desorb‐able” (i.e., mobilizable) P per gram of soil, the water/soil ratio, and the total mass of soil in the zone of interaction (Ahuja et al., 1981; Sharpley et al., 1981). In the context of describing DP mobilization, the term “desorption” is frequently used but potentially misleading. Unless otherwise defined, desorption implies that reactions between orthophosphate and adsorption surfaces (e.g., clays) control DP mobilization. While desorption may have a broader context that can include chemical dissolution and physical processes (e.g., diffusion‐controlled desorption) (Koopmans et al., 2004; Sharpley et al., 1981), the term is rarely explicitly defined.

**FIGURE 2:**
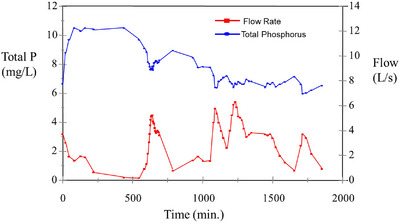
Within‐storm variation in total phosphorus concentration and flow from a field in the Darnum region of West Gippsland, Australia, on November 6, 1994. Adapted from Nash and Murdoch (1997).

A difficulty in conceptualizing DP mobilization using only a mixing layer model is that it appears inconsistent with many field (i.e., >2 ha) studies measuring DP concentrations (Nash, 2002; Nash & Barlow, 2009; Nash et al., 2007; Nash et al., 2021) and studies using flumes in the absence of infiltration (Barlow, 2003; Doody et al., 2006). A mixing layer model would suggest that an initial peak in DP concentrations should be followed by a marked decline as readily accessible sources of DP are exhausted. However, as demonstrated in a study where runoff DP was 90% of TP (Figure 2), a rapid increase in P is often followed by a gradual decline (Nash & Murdoch, 1997). Such a within‐storm concentration profile is consistent with DP mobilization having two phases:

1. At the start of an event, high rates of DP mobilization are attributable to DP release (e.g., by dissolution, desorption) and entrainment (e.g., by advection) that decline as readily accessible supplies of P are exhausted (i.e., P mobilization is supply limited).
2. As the event progresses, a second phase of P mobilization becomes more prominent, where DP release may be rapid but entrainment is slow, resulting in DP being mobilized at a near constant rate for the remainder of the event.

**FIGURE 3:**
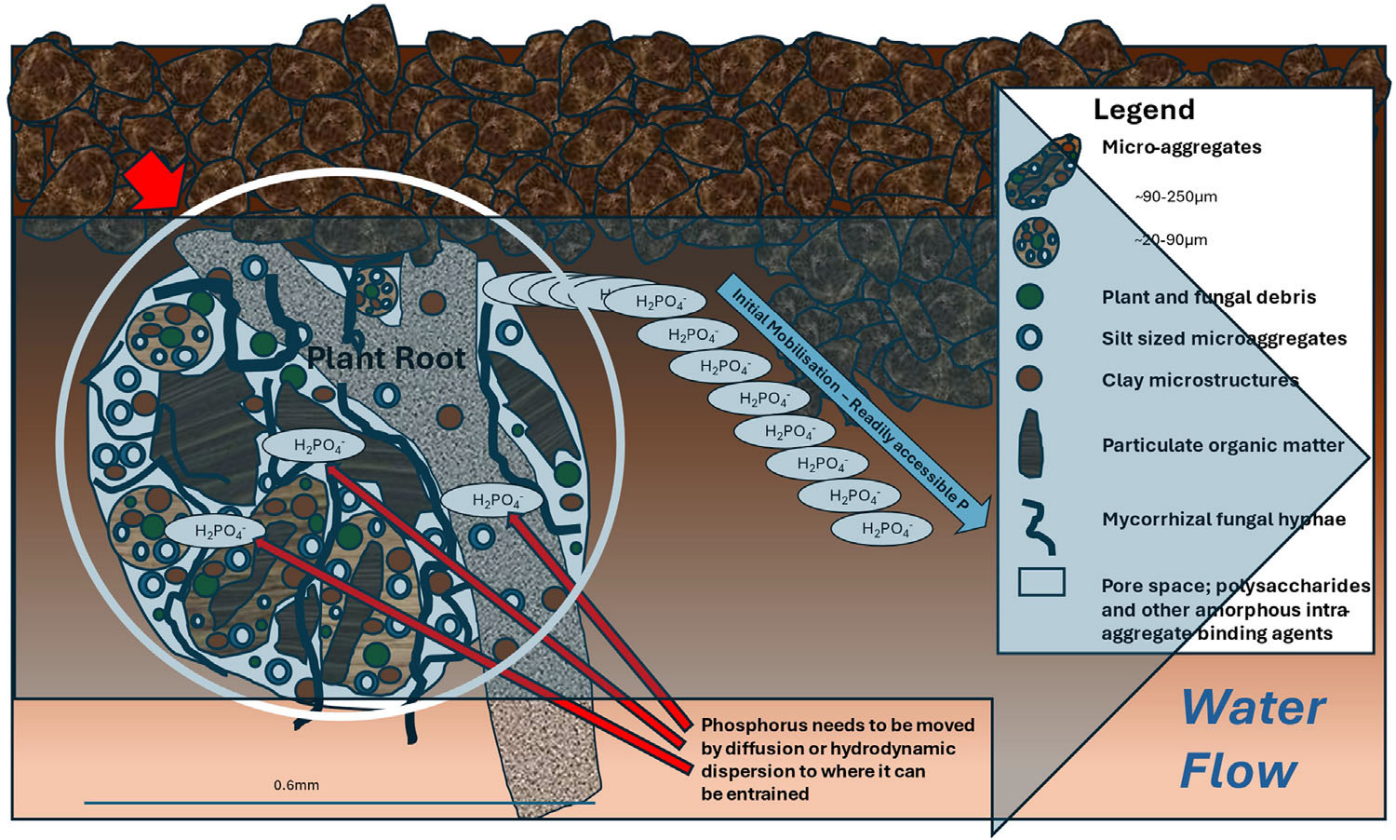
Field‐scale processes contributing to dissolved P mobilization. Adapted from McCarl et al. (2007); Nash et al. (2021).

Advective transport of dissolved pollutants such as P early in a storm or irrigation event is well documented (Wallach, 1991). Whether DP is released through desorption or chemical dissolution of readily accessible orthophosphate is of little consequence, as both would be rapid in comparison to the length of many drainage events. Entrainment following either process is also likely to be rapid and, as a result, stores of readily accessible P quickly depleted. The second phase of the process is more sustained and capable of mobilizing DP at a near constant rate for long periods. The most likely explanation is that diffusion and hydrodynamic dispersion are responsible for entraining DP released (e.g., by desorption or dissolution) from the significant stores of P that are known to reside in aggregates and other surface soil materials (e.g., plant detritus) (see Figure 3). In either case, the distance individual DP components must traverse before entrainment is complete is likely to provide a throttle (i.e., restriction) on mobilization rates. Additional evidence supporting a two phase model of DP mobilization includes the following:

1. Rainfall simulation studies where water was applied on successive days but DP concentrations did not decline in a manner that was consistent with a mixing layer model (Nash et al., 2021; Sharpley, 1995).
2. Field studies where strong relationships between overland flow volumes and flow‐weighted DP concentrations for individual events have not been found (Nash et al., 2000; Nash, Clemow, et al., 2005).

Some observations of DP exports may appear to be at odds with a two‐phase model of DP mobilization. In dry soils, infiltrating water may transport DP from the first phase of mobilization into the soil (i.e., by vertical matrix flow [Nash et al., 2002]) away from where it would be conveyed offsite (Wallach et al., 2001). Conversely, in fields where sub‐surface flows (e.g., interflow) (Snyder & Woolhiser, 1985) re‐emerge, they can add additional P to transport pathways (Govindaraju, 1996). It follows that factors affecting soil hydrology, such as soil water content (i.e., antecedent soil moisture) and soil infiltration characteristics, have been shown to influence P concentrations in runoff (Ahuja, 1990; Buda et al., 2009; Kleinman et al., 2006; Needelman, 2002; Snyder & Woolhiser, 1985; Srinivasan et al., 2007; Vadas et al., 2008) and are likely responsible for some differences in between‐storm variation (i.e., event DP concentrations) in field studies. However, mass transfer is not the only way field hydrology can affect DP exports.

The organic‐enriched layer near the soil surface is probably a reasonable approximation of a mixing layer in which DP mobilization is initiated. Water moving through that layer is functionally indistinguishable from true overland flow (Nash et al., 2002) and affects both DP mobilization and transport. Being exacerbated by factors that suppress infiltration (Alderfer & Robinson, 1947; Drewry, 2006; Houlbrooke et al., 2009), such near‐surface interflow manifests itself in the form of spongy, saturated surface soil that exudes water on compression. Its existence is supported by the observation that surface drainage often continues after water applications, and true overland flow, have ceased. Clearly, understanding the relationships between DP mobilization and hydrology is fundamental to developing robust and effective mitigation tools and strategies.

## 3 PHOSPHORUS INDICES AND ASSESSING THE RISK OF PHOPSPHORUS EXPORTS

Phosphorus indices have been widely used to assess the potential for P to be exported from agricultural fields to surface water (Bechmann et al., 2007; Daniel et al., 2004; Goulet et al., 2006; Lemunyon & Gilbert, 1993; Mallarino et al., 2005; Reid et al., 2018). They often incorporate a local assessment of the source and transport factors (e.g., topography, hydrology, P amendment application, and cropping method) affecting P exports and combine them, usually with an impact factor, into a single metric or risk assessment (Buczko & Kuchenbuch, 2007; McDowell et al., 2001; Nelson & Shober, 2012; Sharpley et al., 2017). A strength of this approach has been the flexibility to integrate research data and experiential knowledge (i.e., professional judgment) into tools that can be used to guide P export mitigation in particular regions. They have proven especially useful for identifying areas where source and transport factors overlap. These areas are commonly referred to as critical source areas (CSAs) (McDowell & Srinivasan, 2009; McDowell et al., 2024) or “risk areas.”

CSAs are important because they are assumed to generate the highest P loads per unit area. Therefore, focusing on those areas is generally a cost‐efficient way to reduce the downstream impacts of agriculture. In recent New Zealand legislation, a CSA was more broadly defined as a landscape feature such as a gully, swale, or depression that (a) accumulates runoff from adjacent land, and (b) delivers, or has the potential to deliver, one or more contaminants to one or more rivers, lakes, wetlands, or drains, or their beds (regardless of whether there is any water in them at the time) (New Zealand Government, 2020). The definition of CSAs in this way is particularly relevant for DP. Unlike PP that can be removed from water by physical means (e.g., buffer strips, retention structures, and wetlands) if only temporarily, once entrained there are few options to remove DP from water. It is therefore important to mitigate DP exports through reduced mobilization rather than reduced transport.

Table 1 presents a summary of some of the source and transport factors incorporated into various P indices. It is noticeable that there is a heavy emphasis on P amendments, primarily manures and fertilizers, and factors that may affect the availability of the P they contain. The P availability with time from most P amendments is likely to follow a decay function that eventually plateaus to a new baseline that is a “legacy” of current and previous management. In the short term, the rates at which P is applied in amendments and application methods (e.g., surface application) would often be expected to overshadow (e.g., swamp) the effects of the preexisting soil fertility. It follows that in such cases indices should predict overall P export potential quite well and be useful for mitigating incidental P exports, especially where appropriate management interventions are applied (e.g., the 4R concept: to apply the right source of nutrients, at the right rate, at the right time and in the right place (Bruulsema, 2018; Johnston & Bruulsema, 2014).

**TABLE 1:** Some factors are used in current P indices to estimate the downstream risk of P exports from agriculture. Adapted from Buczko and Kuchenbuch (2007).

Factor	Significance	Incorporation in P index
**Source factors**		
Soil test phosphorus (STP)	DP concentration in runoff is related to STP; TP is related to STP (P in eroded material)	Essential component in P indices; agronomic STP methods used
Manure‐application rate	Direct losses of soluble P components; increase P levels in soil; manure has lower N:P ratios than crop needs.	Essential component in P indices
Manure‐application method	Great influence on direct losses (surface broadcast > ploughed under > injection)	Essential component in P indices (mostly in conjunction with timing)
Manure‐application timing	Great influence on direct losses (the sooner it rains after application, the greater the risk of P loss)	Essential component in P indices (mostly in conjunction with method)
Fertilizer‐application, method, timing	Similar as for manure	Essential component in P indices
Degree of P saturation	Some studies indicate that DPS is better related to DP in runoff than STP	In some P indices
P in plant residues	Leaching of P from plant residues promoted by repeated freeze‐melting cycles	In a few P indices (Norway, Canada)
**Transport factors**		
Water erosion	TP loss (especially PP) strongly related to erosion	Essential component in P indices
Wind erosion	Similar to water erosion, but delivery pathways very difficult to predict	Considered only in very few P indices
Surface runoff	Transport of dissolved P	Essential component in P indices
Irrigation runoff	Improper irrigation management can induce runoff and erosion	Component in some P indices
Subsurface flow	Important in sandy and macroporous soils; enhanced by tile drains	Component in most recent P indices
Contributing distance	The nearer a field is to a receiving water, the higher the risk of P loss	Essential component in most P indices
Riparian‐buffer width	The greater the buffer width, the more effective the filtering of PP	Component in most P indices, often as “modified connectivity”
Sensitivity of receiving waters	Surface waters react differently to P input	Considered only in very few P indices

Abbreviations: DP, dissolved phosphorus; DPS, degree of P saturation; PI, phosphorus index; PP, particulate phosphorus; TP, total phosphorus; STP, soil test phosphorus.

It is also notable in Table 1 that in P indices, STP is used to infer the potential for both DP and PP exports. Mobilization of PP is primarily a physical process that commences with detachment of fine particles (e.g., sediments) and associated P from aggregates and other soil materials. The term “erosion” is used to refer to a process where mobilization (i.e. detachment) is followed by entrainment of particulate materials in flowing water. Detachment is facilitated by physical processes, including raindrop impact, cultivation, cattle treading damage, and flowing water, and physico‐chemical processes, such as slaking and dispersion (Davies & Payne, 1988; Leeper & Uren, 1997). The capacity of water to transport particulate materials is related to the kinetic energy of the water (Shainberg et al., 1994). Factors that increase water velocity (e.g. slope) and turbulence (e.g. obstructions) generally increase detachment and transport rates (Kelley, 1983; Smith & Wischmeier, 1962; Wischmeier & Smith, 1978). While notoriously difficult to model, erosion and PP exports from agriculture are increasingly well handled in the more modern P indices (Reid et al., 2018; Weld, 2022). However, there is little to suggest that mitigation measures targeting the physical aspects of PP mobilization will be similarly successful for DP.

The development of P indices has been an iterative process with a trend toward increased compartmentalization to address specific conceptual and actual deficiencies (Sharpley, Beegle, et al., 2012), and regulatory requirements (Nelson & Shober, 2012; U.S. Department of Agriculture—National Nesource Conservation Service, 2012). For example, one of the original P indices used a two‐dimensional matrix to relate field characteristics to P export potential without consideration of whether offsite transport was likely (Lemunyon & Gilbert, 1993). The P index was subsequently modified and the product of the source and transport factors used to assess edge‐of‐field export potential (Gburek et al., 2000). More recently, in response to a mistaken perception that controlling erosion effectively mitigates the impact of agriculture on receiving waters (Baker et al., 2014), many P indices have moved from reporting only TP export potential to reporting DP and PP potential separately (see for example, Weld, 2022).

Despite the improvements in P indices, some challenges remain. Dissolved P export potential is usually assessed using “surface runoff” and an “extraction coefficient” defined as the slope of the linear regression between STP and runoff P (Vadas et al., 2005). Given the scale (e.g., <10m^2^) and duration (e.g., <1 h) of simulation studies, they are only likely to reflect the first phase of DP mobilization (Nash et al., 2021). Moreover, the pretreatment of the samples (e.g., drying, sieving, and repacking soil) may not be representative of field conditions.

The difficulties in using STP for estimating DP exports have been well demonstrated by a study that used edge of field monitoring of cropland to examine relationships between the Mehlich 3 soil P test and DRP in both surface runoff (i.e., presumably overland flow) and tile drainage (Osterholz et al., 2020). Mehlich 3 (i.e., 0–200 mm) explained 19% (i.e., *R* ^2^) of the variation in runoff and 32% of the variation in surface flow and tile drainage. The equivalent figures for 0–50 mm sampling were 32% and 44%. The authors noted that STP variability between sites within fields and between sampling times affected those relationships. These results are consistent with modeling of between‐storm variation in Australia (Nash, Clemow, et al., 2005). In that study of a rainfed site, the effect of year (i.e., systematic P exports) was poorly related to the increasing Olsen P (e.g., 17–57 mg/kg).

For P indices there are also challenges in the way they balance P attributable to management activities (i.e., incidental sources) as versus legacy P sources. Dissolved P mobilization from an amendment can be significantly greater and functionally different from that occurring from legacy P. Some indices address this by including the quantity of water‐soluble P being applied (DeLaune et al., 2004a, 2004b). However, if a P index is being used to delineate CSAs, it is likely to focus on high‐transport areas, whereas systematic DP mobilization from legacy P might be surreptitiously occurring elsewhere (e.g., on flatter land). This may help explain why a review of studies of CSAs from 1990 to 2023 shows that the proportion of the annual contaminant load coming from a CSA decreases from field to farm to catchment scale as our ability to map CSAs decreases and other processes (e.g., groundwater influx or in‐stream attenuation) become more important (McDowell et al., 2024). It may also help explain why efforts to lessen PP exports from cropland led to higher DP (i.e., systematic legacy) exports through the combination of P stratification and tile drainage in the Lake Erie catchment (Baker et al., 2017; Daloğlu et al., 2012).

## 4 A PHOSPHORUS INDEX STRUCTURE THAT REFLECTS LEGACY P AND PROVIDES FOR FUTURE DEVELOPMENT

While P indices have their challenges for assessing the P export potential, they also have their place. But as the example of Lake Erie demonstrates, there is a pressing need to better account for DP and PP exports separately. Similarly, there is a need to account for incidental and systematic P exports separately, especially where short‐term P additions may initially overshadow systematic exports but have a lasting legacy. More complex, integrated tools are needed to guide P export mitigation in the future (Reid et al., 2018; U.S. Department of Agriculture—National Resource Conservation Service, 2017).

Bayesian networks, also called Bayesian belief networks, are an alternative approach to the numerical conditioning (i.e., using weighting factors) currently used in some indices. They can generate numerical outcomes similar to current indices, but also illustrate the variability of the outcomes and the interrelationships between system components. Bayesian networks are based on conditional probability theory. They provide a graphical representation of “cause and effect” relationships with the strength of the interdependencies (causal links) represented as conditional probabilities (Jensen & Nielsen, 2007; Korb & Nicholson, 2004; Pourret et al., 2008). Bayesian networks have already been used to develop conceptually sound, mechanistic (i.e., quasi process) representations of nutrient export processes in agricultural systems (Lucci et al., 2014; McDowell et al., 2009; Nash & Hannah, 2011; Nash et al., 2010; Nash et al., 2013). The nodes represent variables (discrete or continuous) and directed links (also called arcs, which pass from the parent node to the child node) are used to represent dependencies between variables. Dependencies are quantified by conditional probability distributions that are associated with each node. Bayesian networks can be developed using similar information to the original P indices: (a) direct data analyses/tabulation of observed frequencies (e.g., for probability of rainfall); (b) elicitation of expert opinion; (c) Monte Carlo simulations where deterministic relationships are known; and, where sufficient data are available, (d) machine learning techniques. Importantly, the output from Bayesian networks can be integrated (i.e., exported) into other modeling platforms.

Irrespective of the platform used, for the foreseeable future STP will underpin assessments of DP mobilization from legacy (i.e., systematic) sources. On its own, STP has severe limitations for estimating DP mobilization potential. In part, that is due to factors (e.g., spatial and temporal variation) that are difficult to quantify or improve. However, the conceptual model of DP mobilization presented earlier can be used to improve the assessment of P export potential.

**FIGURE 4:**
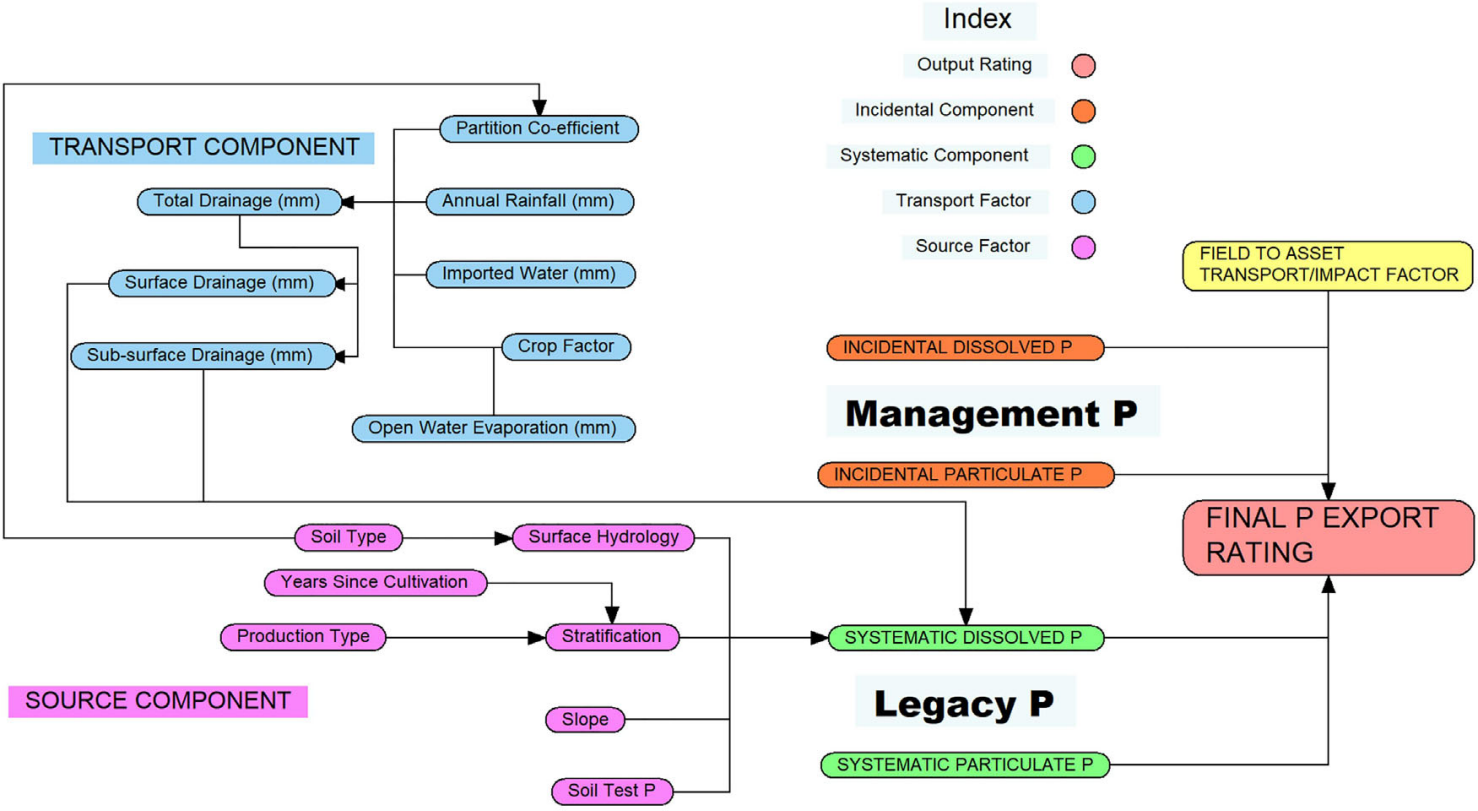
A flow diagram illustrating some potential relationships that could be used to infer dissolved phosphorus export potential from legacy sources.

A rudimentary flow diagram that focuses on factors that affect the DP mobilization potential from legacy sources (i.e., SYSTEMATIC DISSOLVED P) is presented in Figure 4. The TRANSPORT COMPONENT in Figure 4 is based on conservation of mass and is included primarily to reflect the importance of soil hydrology, as well as the need to adapt P indices to the regions in which they will be used. For example, frozen soil affects water infiltration, leading to increased overland flow during periods of snowmelt (Li et al., 2011; Wu & Fang, 2024). Consequently, overland flow volumes from snow often exceed those from rainfall and the duration of surface runoff is longer (Granger et al., 2011; Little et al., 2007; Mohammed et al., 2018), providing increased opportunities for DP mobilization (Liu et al., 2019).

In the conceptual model, DP mobilization was initiated at or near the soil surface. It follows that conditioning (i.e., modifying) STP to allow for soil stratification would improve estimates of DP export potential (i.e., risk). Such conditioning could be best accomplished using a probability density function rather than a single value so as not to overlook the influence of outlying values and their compounding effects. Tillage (i.e., ploughing and destratification) has been shown to significantly lower DP concentrations at the soil surface and therein systematic DP mobilization potential (Mathers & Nash, 2009; Nash et al., 2007; Nash et al., 2015; Watkins et al., 2012). It follows that “production type” (e.g., crop, intensive grazing) and “years since cultivation” both affect “stratification” and should be used to guide the development of a locally appropriate density function.

A factor that affects P mobilization but is rarely represented in current P indices in relation to DP mobilization is “slope.” The conceptual model suggests that the bulk of DP exports occur in the second phase of mobilization, where the time for entrainment places a limitation (i.e., throttle) on DP mobilization rates. That suggests that DP mobilization is largely independent of flow rate and more DP will be mobilized on lower slopes where the surface soil and water interact for longer periods. In many systems that interaction will occur as near‐surface interflow. Unfortunately, those are also the areas where drains may be used that link areas of high DP mobilization potential to transport pathways.

The importance of hydrology in assessing P export potential should not be underestimated. In Figure 4 “soil type” informs the “partition coefficient” (i.e., between “subsurface drainage [mm]” and “surface drainage [mm]”). In affecting transport pathways, “soil type” affects SYSTEMATIC DISSOLVED P. The effects of hydrology on DP mobilization are likely to vary between regions and systems, emphasizing the need for incorporating local knowledge into P indices. Such knowledge includes the introduction of new management systems (e.g., tile drains).

## 5 CONCLUDING DISCUSSION

The use of P amendments has been essential in the development of modern agriculture. These amendments have generally been used in surplus (i.e., positive farm‐gate balances), so their legacy has been higher soil fertility (i.e., STP). That residual (i.e., legacy) P has increased exports of both PP and the more bioavailable DP.

Phosphorus indices have been an important tool for addressing the “legacy” P cycling in modern agricultural systems. However, as the Lake Erie experience demonstrates, there is a need to consider PP and DP separately. More so because, unlike PP, once entrained DP can be difficult to remove from drainage unless it attaches to particulate materials during transport. There is a similar need to separately consider the risks attributable to systematic (i.e., legacy) and incidental factors since the short‐term management of P amendments can initially overshadow their residual (i.e., legacy) effects.

Soil tests are currently used in P indices to estimate the risks associated with DP from legacy sources. By themselves, soil P tests have challenges for estimating the risk of unacceptable DP exports. However, a conceptual understanding of DP mobilization facilitates the use of local information to condition soil test data for the purposes of assessing export potential.

It would appear that there are two phases of DP mobilization from legacy sources. Early in a drainage event, DP is released from near the soil surface by desorption and dissolution and quickly entrained in the water that ultimately transports it offsite. As the event progresses and readily accessible supplies of DP are exhausted, there is resupply from less accessible sources (e.g., DP contained in plants, detritus, and aggregates). This second phase of mobilization is limited by the time it takes for diffusion and hydrodynamic dispersion to entrain DP and provides a physical limitation (i.e., throttle) on the rate of DP mobilization. Importantly, since most P exports occur in larger (i.e., longer) events, on an annual basis the DP load from the second phase of mobilization predominates. These processes are not well represented in P indices at present.

Conditioning the output from STP data based on “stratification” and factors that affect the time of surface soil and water interaction, such as “slope,” would appear to be useful improvements to P indices. However, the effects of hydrology are equally important. As the Lake Erie example has shown, changing hydrology can compound the risks associated with other, apparently minor changes in an agricultural system. In time it may be possible to develop risk profiles for DP using tracing technologies (Nash & Halliwell, 2000; Nash, Leeming, et al., 2005). For the present, using STP conditioned using local knowledge will have to suffice.

## AUTHOR CONTRIBUTIONS

**D. M. Nash**: Conceptualization; writing—original draft; writing—review and editing. **R. W. McDowell**: Conceptualization; writing—review and editing. **P. J. A. Kleinman**: Conceptualization; writing—review and editing. **P. A. Moore**: Conceptualization; writing—review and editing. **J. M. Duncan**: Conceptualization; writing—review and editing. **P. M. Haygarth**: Conceptualization; writing—review and editing. **D. R. Smith**: Conceptualization; writing—review and editing. **A. Iho**: Conceptualization; writing—review and editing.

## CONFLICT OF INTEREST STATEMENT

The authors declare no conflicts of interest.
